# Supplementary material for: Multi‐dataset identification of innovative feature genes and molecular mechanisms in keratoconus
Source: J Cell Mol Med. 2024 Sep 19;28(18):e70079. doi: 10.1111/jcmm.70079 (PMC11412914; doi:10.1111/jcmm.70079)
Supplement: Supplementary file 2 — Material S2. [file JCMM-28-e70079-s002.zip › 7.GSEA analysis/1.ARL11/KEGG_PANTOTHENATE_AND_COA_BIOSYNTHESIS.html]

Details for gene set KEGG\_PANTOTHENATE\_AND\_COA\_BIOSYNTHESIS[GSEA]

|  || Dataset | ARL11.group.cls#h\_versus\_l |
| Phenotype | group.cls#h\_versus\_l |
| Upregulated in class | h |
| GeneSet | KEGG\_PANTOTHENATE\_AND\_COA\_BIOSYNTHESIS |
| Enrichment Score (ES) | 0.30248433 |
| Normalized Enrichment Score (NES) | 0.79184455 |
| Nominal p-value | 0.7710843 |
| FDR q-value | 1.0 |
| FWER p-Value | 1.0 |
Table: GSEA Results Summary

  

Fig 1: Enrichment plot: KEGG\_PANTOTHENATE\_AND\_COA\_BIOSYNTHESIS      
 Profile of the Running ES Score & Positions of GeneSet Members on the Rank Ordered List

  

| SYMBOL | TITLE | RANK IN GENE LIST | RANK METRIC SCORE | RUNNING ES | CORE ENRICHMENT || 1 | BCAT1 | na | 876 | 0.403 | 0.2008 | Yes |
| 2 | DPYD | na | 3337 | 0.225 | 0.2561 | Yes |
| 3 | DPYS | na | 4971 | 0.167 | 0.3025 | Yes |
| 4 | VNN2 | na | 9372 | 0.069 | 0.2146 | No |
| 5 | PANK1 | na | 9716 | 0.064 | 0.2405 | No |
| 6 | PPCS | na | 11197 | 0.045 | 0.2232 | No |
| 7 | ENPP3 | na | 12321 | 0.033 | 0.2094 | No |
| 8 | PANK2 | na | 13241 | 0.025 | 0.1969 | No |
| 9 | PPCDC | na | 15631 | 0.006 | 0.1317 | No |
| 10 | COASY | na | 16890 | -0.002 | 0.0968 | No |
| 11 | PANK3 | na | 17239 | -0.005 | 0.0896 | No |
| 12 | BCAT2 | na | 17506 | -0.007 | 0.0858 | No |
| 13 | ENPP1 | na | 23556 | -0.057 | -0.0568 | No |
| 14 | PANK4 | na | 28382 | -0.140 | -0.1172 | No |
| 15 | VNN1 | na | 31674 | -0.247 | -0.0737 | No |
| 16 | UPB1 | na | 32517 | -0.288 | 0.0637 | No |
Table: GSEA details [plain text format]

  

Fig 2: KEGG\_PANTOTHENATE\_AND\_COA\_BIOSYNTHESIS      
 Blue-Pink O' Gram in the Space of the Analyzed GeneSet

  

Fig 3: KEGG\_PANTOTHENATE\_AND\_COA\_BIOSYNTHESIS: Random ES distribution      
 Gene set null distribution of ES for **KEGG\_PANTOTHENATE\_AND\_COA\_BIOSYNTHESIS**
